# Patella tendon autograft use and A‐shaped femoral notch are associated with more anterior and proximal femoral tunnel placement during anterior cruciate ligament reconstruction

**DOI:** 10.1002/jeo2.70694

**Published:** 2026-03-22

**Authors:** Sahil Dadoo, Koji Nukuto, Efstathios Konstantinou, Jumpei Inoue, Jacob Hartline, Jonathan D. Hughes, James J. Irrgang, Volker Musahl

**Affiliations:** ^1^ Department of Orthopaedic Surgery, UPMC Freddie Fu Sports Medicine Center University of Pittsburgh Pittsburgh Pennsylvania USA; ^2^ Department of Physical Therapy, School of Health and Rehabilitation Sciences University of Pittsburgh Pittsburgh Pennsylvania USA

**Keywords:** ACL reconstruction, femoral notch, femoral tunnel, patellar tendon autograft, tunnel malposition

## Abstract

**Purpose:**

The purpose of this study was to evaluate the impact of surgical and anatomical variables, including graft type, femoral notch width and shape, posterior cruciate ligament (PCL) width and lateral femoral condyle (LFC) diameter, on femoral tunnel position during anterior cruciate ligament reconstruction (ACLR).

**Methods:**

All consecutive patients who underwent primary ACLR between 2017 and 2021 were retrospectively reviewed. Femoral tunnel position was evaluated in the posterior‐to‐anterior (P‐A) and proximal‐to‐distal (P‐D) directions using the quadrant method. Surgical and anatomical variables (including patient demographics, ACLR graft type, femoral notch width and shape, PCL width and LFC diameter) were compared between the most anterior and proximal quartiles of femoral tunnel positions to the rest of the cohort to identify variables associated with anterior and proximal femoral tunnel placement. Statistical tests included independent *t* test, Mann–Whitney *U* test and *χ* ^2^ test as appropriate, with significance set to *p* < 0.050.

**Results:**

A total of 372 patients (mean age: 25 years; 190 [51%] females) were included for analysis. Compared to the non‐anterior group, the anterior quartile group had a higher proportion of bone–patellar tendon–bone (BPTB) autografts (38% BPTB vs. 22% BPTB; *p* = 0.002) and lower proportion of quadriceps tendon (QT) autografts (29% QT vs. 50% QT; *p* = 0.002). Compared to the non‐proximal group, the proximal quartile group had a higher proportion of A‐shape femoral notches (53% vs. 39%; *p* = 0.016) and smaller LFC diameter (30.4 vs. 31.3 mm; *p* = 0.048). Female patients additionally had 1.9 times higher odds of proximal femoral tunnel placement compared to male patients.

**Conclusion:**

BPTB autograft use was associated with more anterior femoral tunnel placement, while an A‐shape femoral notch, smaller LFC diameter and female sex were associated with more proximal femoral tunnel placement during primary ACLR. For clinical relevance, these data may be useful to surgeons to identify demographic, surgical and anatomical variables that may influence femoral tunnel position during primary ACLR.

**Level of Evidence:**

Level IV, case series.

## Graphical abstract

Patella tendon autograft use and an A‐shaped femoral notch were associated with more anterior and proximal femoral tunnel placement, respectively, during primary ACL reconstruction.

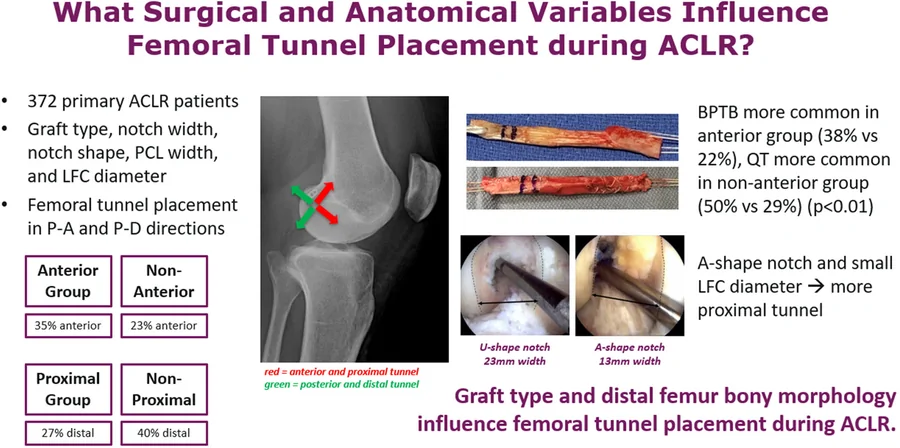

**Abbreviations**
ACL: anterior cruciate ligament
ACLR: anterior cruciate ligament reconstruction
AM: anteromedial
BPTB: bone–patellar tendon–bone
EMR: electronic medical record
HT: hamstring tendon
ICC: intraclass correlation coefficients
LET: lateral extra‐articular tenodesis
LFC: lateral femoral condyle
MRI: magnetic resonance imaging
OA: osteoarthritis
OR: odds ratio
P‐A: posterior‐to‐anterior
PCL: posterior cruciate ligament
P‐D: proximal‐to‐distal
QT: quadriceps tendon

## INTRODUCTION

The goal of anatomic anterior cruciate ligament (ACL) reconstruction (ACLR) is to restore knee laxity and kinematics by replicating the native anatomy of the intact ACL [8, 34]. Previous biomechanical and clinical studies have demonstrated anatomic ACLR results in improved rotatory knee laxity, reduced ACLR failure rates and lower rates of subsequent meniscus injury and post‐traumatic knee osteoarthritis (OA) when compared to non‐anatomic ACLR [7, 13, 14, 24, 28, 36, 37]. While several patient‐related risk factors for ACLR failure exist, including age, sex and activity level [10, 38], femoral tunnel malposition is often cited as the most common technical cause of ACLR failure, accounting for over 80% of cases where ACLR failure occurred due to technical or surgical‐related reasons [6, 21, 23].

Despite the importance of anatomic femoral tunnel position on clinical outcomes after ACLR, there is limited literature evaluating factors that may be associated with femoral tunnel position during ACLR. Available evidence suggests smaller femoral notch morphology may predispose a shorter femoral tunnel length, and more distal femoral tunnel placement may be achieved in patients with larger femoral notch volume [15, 16, 17]. However, it remains unknown how surgical and anatomical factors, such as graft type, intra‐articular anatomy and distal femur morphology, may impact femoral tunnel placement during ACLR. As prior literature has demonstrated that even high‐volume surgeons experience inconsistencies in achieved versus intended femoral tunnel position [27], there remains a critical need to understand what variables may influence femoral tunnel position during ACLR.

The purpose of this study was to evaluate the influence of surgical and anatomical variables, including graft type, femoral notch width and shape, posterior cruciate ligament (PCL) width and lateral femoral condyle (LFC) diameter, on femoral tunnel position during primary ACLR. The hypothesis was that a smaller femoral notch width, A‐shape femoral notch, larger PCL width and smaller LFC diameter would be associated with more anterior and proximal femoral tunnel placement during primary ACLR.

## MATERIALS AND METHODS

### Data collection

This retrospective study was approved by the Institutional Review Board at the University of Pittsburgh (No.: STUDY 19030196). All consecutive patients aged ≥14 years old who underwent primary single‐bundle ACLR between 2017 and 2021 by one of six high‐volume (defined as ≥35 ACLR/year) sports medicine fellowship‐trained orthopaedic surgeons were included in this study. All patients who underwent concomitant lateral extra‐articular tenodesis (LET), meniscus root repair, meniscus allograft transplantation, osteotomy or osteochondral autograft or allograft procedures were excluded. Additional exclusion criteria included lack of pre‐operative radiographs or magnetic resonance imaging (MRI) and lack of a true lateral knee radiograph post‐operatively, defined as a distance of <6 mm between posterior femoral condyles to exclude rotational effects [26].

Demographic data including age, sex and body mass index (BMI) were obtained from electronic medical record (EMR). Primary ACLR graft type, including hamstring tendon (HT) autograft, quadriceps tendon (QT) autograft, bone–patellar tendon–bone (BPTB) autograft and allograft, was obtained from operative reports. All patients underwent primary single‐bundle ACLR utilizing anteromedial (AM) portal drilling for creation of the femoral tunnel. The choice of primary ACLR graft type and fixation device was based on surgeon‐specific technique and discretion.

Femoral tunnel position was evaluated on post‐operative true lateral knee radiographs, and femoral notch width and shape, PCL width and LFC diameter were evaluated on pre‐operative MRIs, as outlined below. All imaging measurements were performed by three fellowship‐trained orthopaedic sports medicine surgeons (K.N, E.K, J.I) who were blinded to the patients' medical records.

### Radiographic imaging measurements

#### Femoral tunnel position

Femoral tunnel position was evaluated on strict post‐operative lateral knee radiographs in the posterior‐to‐anterior (P‐A) and proximal‐to‐distal (P‐D) directions using the previously validated quadrant method [4]. The femoral tunnel P‐A position was measured as the distance from the posterior femoral condyle to the centre of the femoral tunnel divided by the total sagittal distance of the femoral condyle along Blumensaat's line, and was recorded as a percentage from 0% to 100% (higher percentage indicating more anterior tunnel placement). The femoral tunnel P‐D position was measured as the perpendicular distance from Blumensaat's line to the centre of the femoral tunnel divided by the total distance from Blumensaat's line to the inferior‐most aspect of the femoral condyle and was recorded as a percentage from 0% to 100% (higher percentage indicating more distal tunnel placement). Based on prior cadaveric studies, the anatomic centre of the femoral ACL footprint was identified at 25% in the P‐A direction and 29% in the P‐D direction (Figure 1) [4].

**Figure 1:**
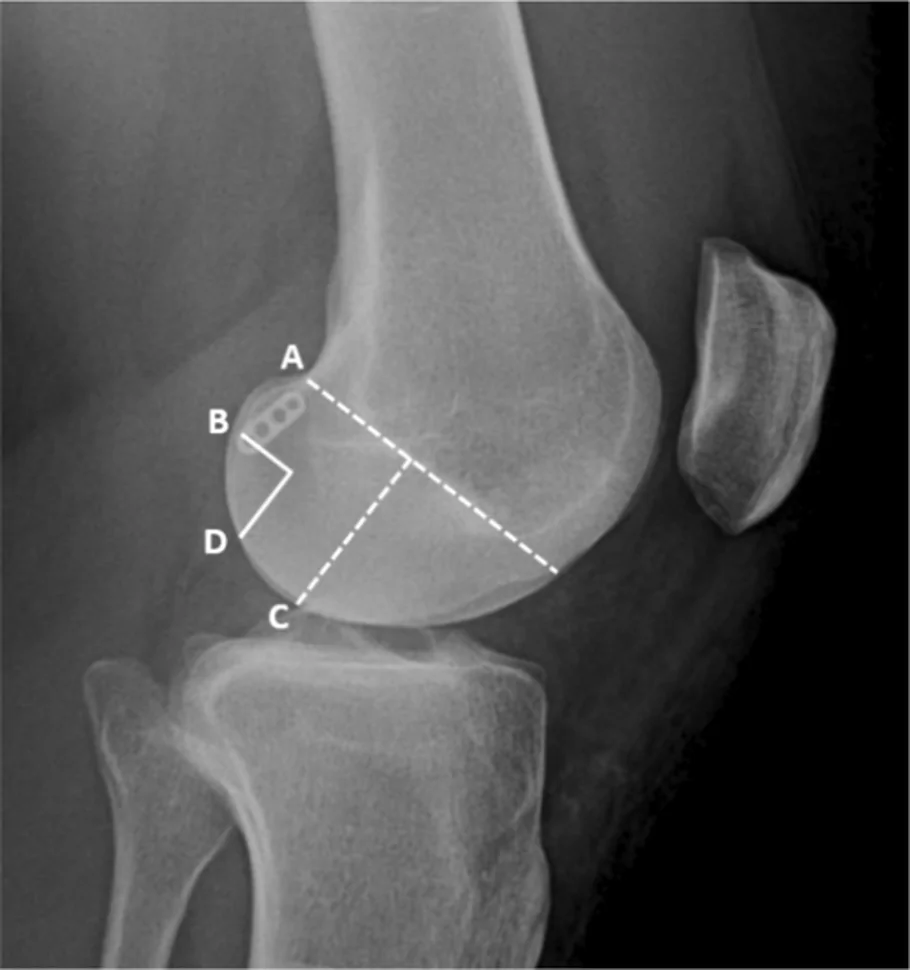
Measurement of femoral tunnel position using quadrant method. Femoral tunnel position in the posterior‐to‐anterior dimension was determined using two lines: Line A was drawn along Blumensaat's line measuring the total sagittal diameter of the lateral femoral condyle, and Line B was drawn parallel to Line A measuring the total sagittal distance from the centre of the femoral tunnel to the posterior wall of the lateral femoral condyle. Tunnel position was determined by the following equation: (Line B)/(Line A) × 100. Femoral tunnel position in the proximal‐to‐distal dimension was determined using two lines: Line C was drawn perpendicular to Line A from Blumensaat's line to the distal contour of the lateral femoral condyle, and Line D was drawn parallel to Line C from the centre of the femoral tunnel to the distal contour of the lateral femoral condyle. Tunnel position was determined by the following equation: (Line D)/(Line C) × 100.

#### Femoral notch width and shape

Femoral notch width was evaluated on the coronal T1 MRI slice in which the popliteal femoral insertion point was most clearly visualized and was measured as the width of the femoral notch at the level of the popliteal groove, parallel to a line tangential to the distal femoral condyles (Figure 2) [33]. Femoral notch width measurements were recorded in millimetres.

**Figure 2:**
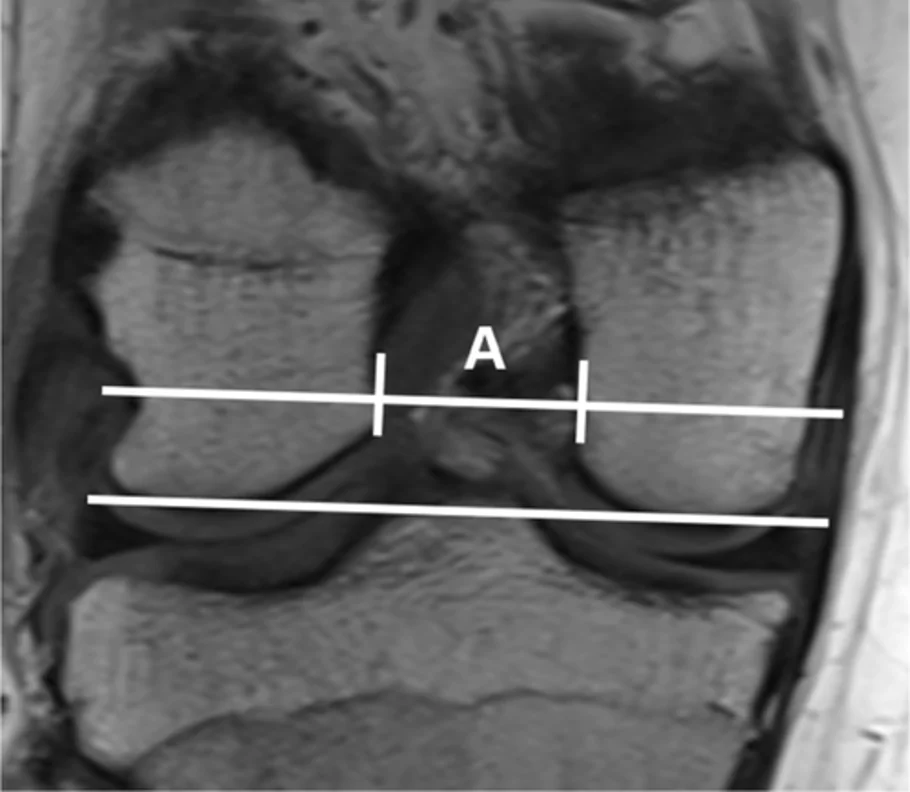
Measurement of femoral notch width using the coronal T1 magnetic resonance imaging slice in which the popliteal femoral insertion point was most clearly visualized, the femoral notch width (A) was measured at the level of the popliteal groove, parallel to a line tangential to the distal femoral condyles and recorded in millimetres.

Femoral notch shape was evaluated using the axial T2 MRI slice in which the entire femoral notch entrance was visualized. Femoral notch shape was grouped as ‘A‐shape’, defined as a notch shape that narrowed from the base to midsection to apex, or ‘U‐shape’, defined as a notch shape in which there was no narrowing from the base to the midsection, based on previous literature definition (Figure 3) [35].

**Figure 3:**
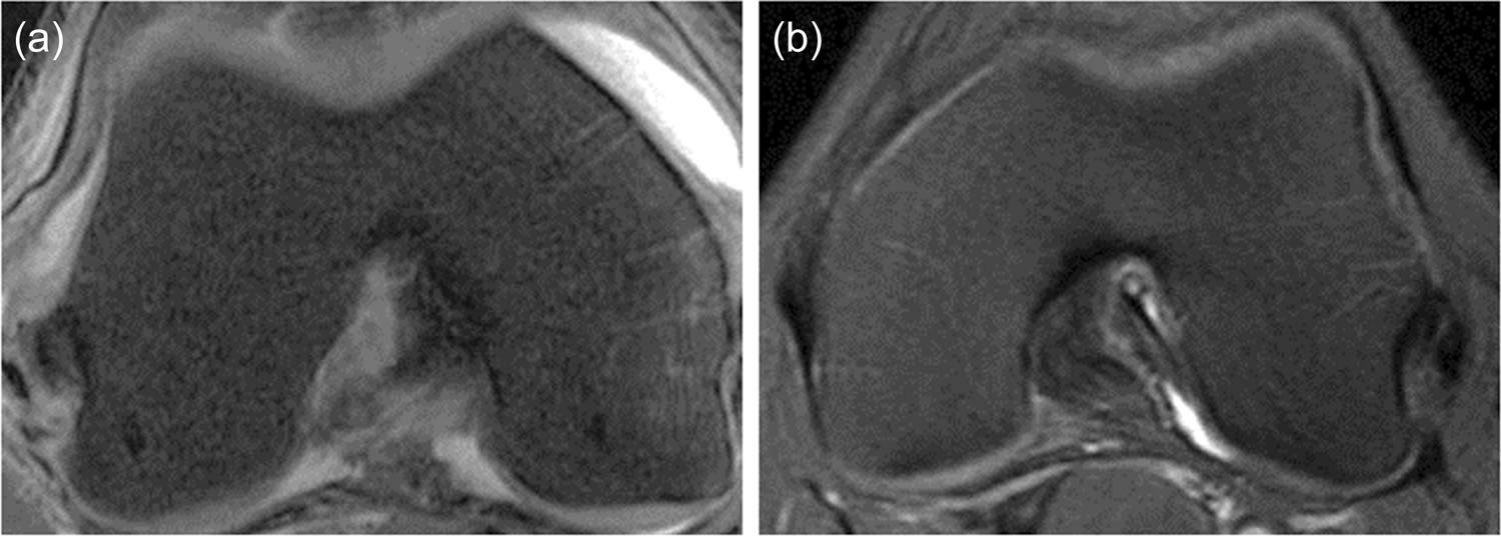
Evaluation of femoral notch shape. Femoral notch shape was evaluated using the axial T2 magnetic resonance imaging slice in which the entire femoral notch entrance was visualized. Femoral notch shape was grouped as (a) ‘A‐shape’, defined as a notch shape that narrowed from the base to midsection to apex, or (b) ‘U‐shape’, defined as a notch shape in which there was no narrowing from the base to the midsection.

#### PCL width

Using the coronal T1 MRI slice in which the PCL was fully visualized, the PCL width was measured as the maximum medial‐to‐lateral width of the PCL and recorded in millimetres (Figure 4) [5].

**Figure 4:**
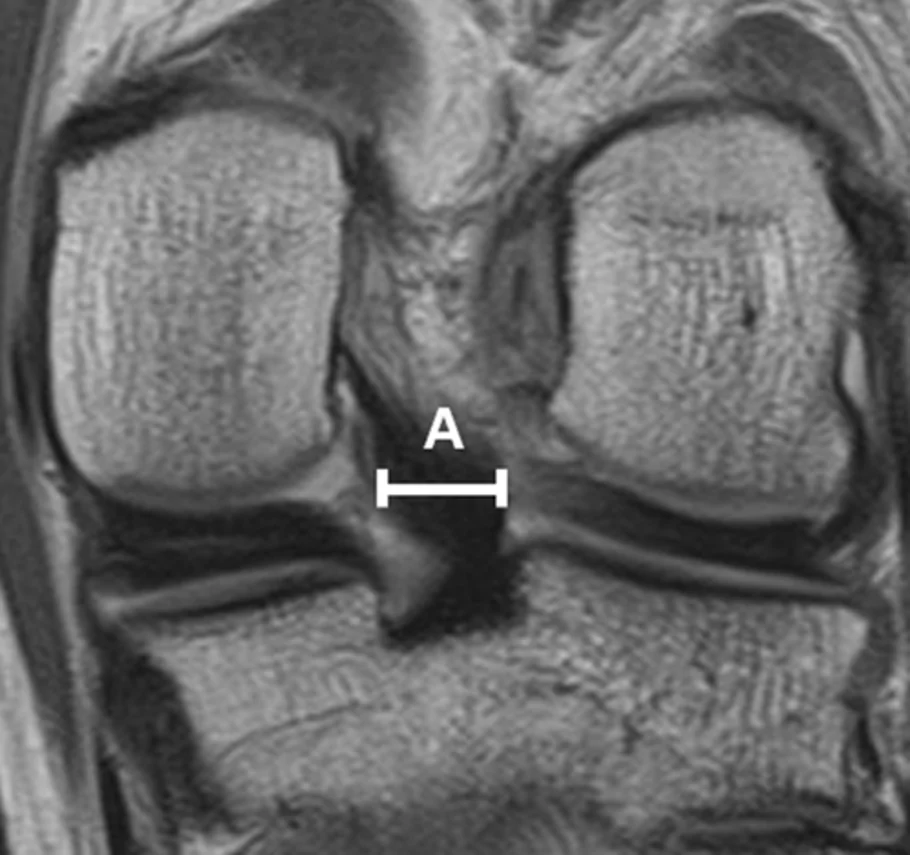
Measurement of posterior cruciate ligament (PCL) width. Using the coronal T1 magnetic resonance imaging slice in which the PCL was fully visualized, the PCL width (A) was measured as the maximum medial‐to‐lateral width of the PCL and recorded in millimetres.

#### LFC diameter

The LFC diameter was measured on the sagittal T1 MRI slice that demonstrated the most posterior projection of the LFC curvature. Three randomly selected points were then drawn along the subchondral contour of the posterior LFC within a defined arc of 10°–135°, and a best‐fit circle was drawn passing through these points. The diameter of the best‐fit circle was recorded in millimetres as the LFC diameter (Figure 5) [11].

**Figure 5:**
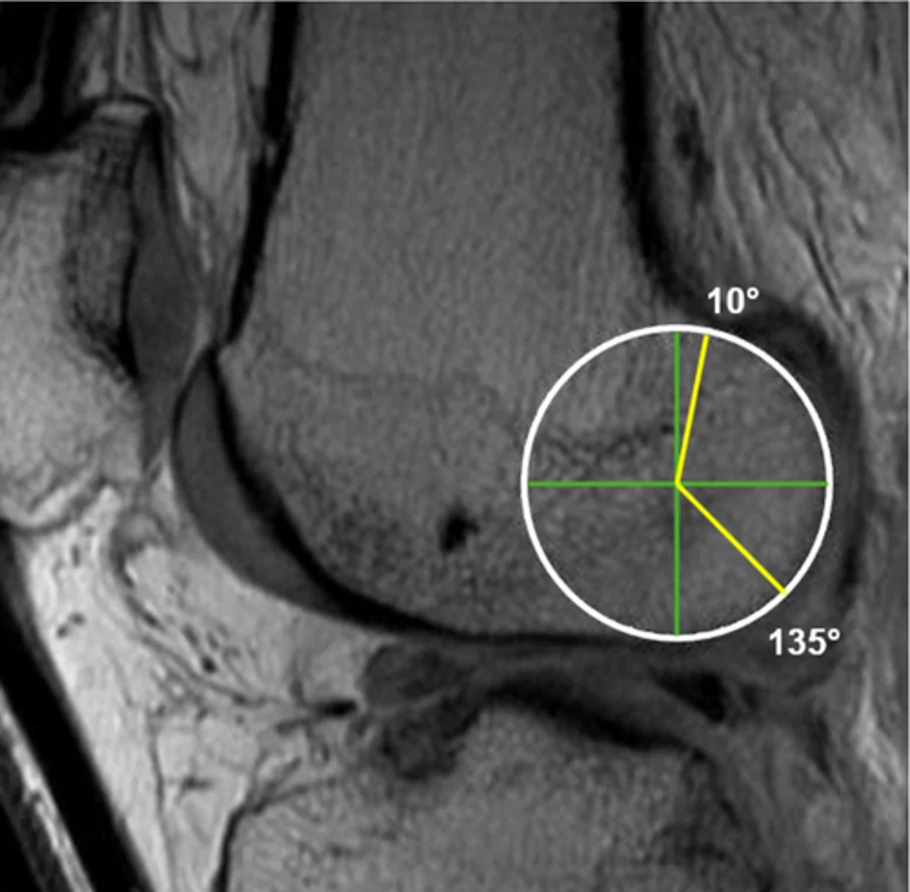
Measurement of lateral femoral condyle (LFC) diameter. The LFC diameter was measured on the sagittal T1 magnetic resonance imaging slice that demonstrated the most posterior projection of the LFC curvature. Three randomly selected points were then drawn along the subchondral contour of the posterior LFC within a defined arc of 10°–135°, and a best‐fit circle was drawn passing through these points. The diameter of the best‐fit circle was recorded in millimetres as the LFC diameter.

### Statistical analysis

Femoral tunnel position was grouped separately in the P‐A and P‐D directions by comparing the most anterior quartile and most proximal quartile of tunnel positions to the remainder of the cohort, respectively. The cutoff value for the most anterior quartile of femoral tunnels was 29% anterior, and the cutoff value for the most proximal quartile of femoral tunnels was 32% distal. Therefore, in the P‐A direction, the ‘anterior quartile’ group included femoral tunnels that were ≥29% anterior, and the ‘non‐anterior’ group included femoral tunnels that were <29% anterior. In the P‐D direction, the ‘proximal quartile’ group included femoral tunnels that were <32% distal, and the ‘non‐proximal’ group included femoral tunnels that were ≥32% distal.

Demographic, surgical and anatomical variables were compared between femoral tunnel position groups in the P‐A and P‐D directions separately. Univariate analyses were performed using independent *t* test or Mann–Whitney *U* test for parametric and non‐parametric data, respectively, and *χ* ^2^ test for dichotomous variables. Logistic regression analysis was performed using all variables significant at the *p* < 0.200 level on univariate analysis to determine independent predictors of anterior and proximal femoral tunnel placement. Significance for all analyses was set to *p* < 0.050.

Intraclass correlation coefficients (ICCs) were calculated using two‐way random effects models for absolute agreement of measurements between raters. In accordance with recommendations previously reported, ICCs were interpreted as poor when below 0.50, moderate when between 0.50 and 0.75, good when between 0.75 and 0.90 or excellent when above 0.90 [18].

## RESULTS

### Total cohort

A total of 704 patients underwent primary single‐bundle ACLR from 2017 to 2021 and were reviewed for inclusion, of which 102 patients were excluded for missing pre‐operative MRI, and 230 patients were excluded for missing true lateral post‐operative knee radiographs. Therefore, after applying exclusion criteria, a total of 372 patients (mean age: 25 ± 11 years; 51% female) were ultimately included for analysis. The distribution of graft types across the cohort included 41 (11%) HT autografts, 162 (44%) QT autografts, 100 (27%) BPTB autografts and 69 (18%) allografts. Among the 162 patients who underwent QT autograft ACLR, 11 (7%) patients had QT autografts with a bone plug, whereas 151 (93%) patients had all soft‐tissue QT autografts. The mean femoral tunnel position in the P‐A direction was 27% anterior (standard deviation [SD]: 7%), and the mean femoral tunnel position in the P‐D direction was 37% distal (SD: 7%). ICCs are summarized in Table 1, and inter‐rater reliability was deemed moderate to excellent for all measurements obtained (Table 1).

**Table 1:** ICCs for inter‐rater reliability for all measurements.

Measurements	ICC^a^
Anatomical measurements	
Femoral notch width	0.88
Femoral notch shape	0.97
PCL width	0.72
LFC diameter	0.98
Femoral tunnel position	
% Anterior (P‐A direction)	0.95
% Distal (P‐D direction)	0.90

Abbreviations: ICC, intraclass correlation coefficient; LFC, lateral femoral condyle; P‐A, posterior‐to‐anterior; PCL, posterior cruciate ligament; P‐D, proximal‐to‐distal.

a ICC was calculated using two‐way random effects models for absolute agreement of measurements between raters. ICCs were interpreted as poor when below 0.50, as moderate when between 0.50 and 0.75, as good when between 0.75 and 0.90, and as excellent when above 0.90.

#### Femoral tunnel P–A position

The mean femoral tunnel P‐A position in the anterior quartile group was 35% ± 6% anterior (range: 29%–62% anterior), while the mean femoral tunnel P‐A position in the non‐anterior group was 23% ± 3% anterior (range: 15%–28%) (*p* < 0.001). There was no significant difference in age (25 vs. 25 years; *p* = 0.969), sex (50% vs. 52% female; *p* = 0.785) or BMI (26 vs. 27 kg/m^2^; *p* = 0.598) between patients with anterior versus non‐anterior femoral tunnels. A significantly higher proportion of BPTB autografts (38% vs. 22% BPTB; *p* = 0.002) and lower proportion of QT autografts (29% vs. 50% QT; *p* = 0.002) were found in the anterior quartile group compared to the non‐anterior group (Table 2). There was no significant difference in femoral notch width (19.9 vs. 19.9 mm; *p* = 0.936), femoral notch shape (44% vs. 41% A‐shape notch; *p* = 0.613), PCL width (10.8 vs. 10.8 mm; *p* = 0.770) or LFC diameter (30.7 vs. 31.2 mm; *p* = 0.285) between patients with anterior versus non‐anterior femoral tunnels (Table 2).

**Table 2:** Comparison of demographic, surgical and anatomical variables between patients with and without anterior femoral tunnel placement.

	Anterior quartile (*n* = 112)	Non‐anterior (*n* = 260)	*p* value
Age (years), mean (SD)	25 (11)	25 (11)	0.969
Sex (female), *n* (%)	56 (50)	134 (52)	0.785
BMI (kg/m^2^), mean (SD)	26 (5)	27 (6)	0.598
Graft type, *n* (%)			**0.002** a
HT	14 (13)	27 (10)	
QT	33 (29)	129 (50)	
BPTB	42 (38)	58 (22)	
Allograft	23 (20)	46 (18)	
Femoral notch width (mm), mean (SD)	19.9 (2.8)	19.9 (2.7)	0.936
Femoral notch shape, *n* (%)			0.613
A‐shape	49 (44)	107 (41)	
U‐shape	63 (56)	153 (59)	
PCL width (mm), mean (SD)	10.8 (1.5)	10.8 (1.5)	0.770
LFC diameter (mm), mean (SD)	30.7 (3.4)	31.2 (3.5)	0.285

*Note*: Bold signifies a statistically significant finding at *p* < 0.050 level.

Abbreviations: BMI, body mass index; BPTB, bone–patellar tendon–bone; HT, hamstring tendon; kg, kilograms; LFC, lateral femoral condyle; m, metre; mm, millimetre; PCL, posterior cruciate ligament; QT, quadriceps tendon; SD, standard deviation.

a Significant differences were observed between QT and BTB at *p* < 0.050 level.

Logistic regression analysis identified that the use of BPTB autograft had 2.1 times higher odds of anterior femoral tunnel placement compared to the use of other graft types (odds ratio [OR]: 2.1, 95% confidence interval [CI]: 1.3–3.4; *p* = 0.003).

#### Femoral tunnel P‐D position

The mean femoral tunnel P‐D position in the proximal quartile group was 27% ± 4% distal (range: 14%–31% distal), while the mean femoral tunnel P‐D position in the non‐proximal group was 40% ± 5% distal (range: 32%–59% distal) (*p* < 0.001). There was no significant difference in age (25 vs. 26 years; *p* = 0.508), sex (45% vs. 54% female; *p* = 0.154), BMI (26 vs. 26 kg/m^2^; *p* = 0.522) or graft type (*p* = 0.580) between patients with proximal versus non‐proximal femoral tunnels (Table 3). A significantly higher proportion of patients with an A‐shape femoral notch was found in the proximal quartile group compared to the non‐proximal group (53% vs. 39% A‐shape notch; *p* = 0.016). Further, a smaller LFC diameter was found in the proximal quartile group compared to the non‐proximal group (30.4 vs. 31.3 mm; *p* = 0.048). There was no significant difference in femoral notch width (19.9 vs. 19.9 mm; *p* = 0.968) or PCL width (10.6 vs. 10.9 mm; *p* = 0.239) between patients with proximal versus non‐proximal femoral tunnels (Table 3).

**Table 3:** Comparison of demographic, surgical and anatomical variables between patients with and without proximal femoral tunnel placement.

	Proximal quartile (*n* = 98)	Non‐proximal (*n* = 274)	*p* value
Age (years), mean (SD)	25 (11)	26 (11)	0.508
Sex (female), *n* (%)	44 (45)	146 (54)	0.154
BMI (kg/m^2^), mean (SD)	26 (6)	26 (5)	0.522
Graft type, *n* (%)			0.580
HT	11 (11)	30 (11)	
QT	41 (42)	121 (44)	
BPTB	31 (32)	69 (25)	
Allograft	15 (15)	54 (20)	
Femoral notch width (mm), mean (SD)	19.9 (2.9)	19.9 (2.7)	0.968
Femoral notch shape, *n* (%)			**0.016**
A‐shape	51 (53)	105 (39)	
U‐shape	47 (47)	169 (61)	
PCL width (mm), mean (SD)	10.6 (1.5)	10.9 (1.5)	0.239
LFC diameter (mm), mean (SD)	30.4 (2.5)	31.3 (3.3)	**0.048**

*Note*: Bold signifies a statistically significant finding at *p* < 0.050 level.

Abbreviations: BMI, body mass index; BPTB, bone–patellar tendon–bone; HT, hamstring tendon; kg, kilograms; LFC, lateral femoral condyle; m, metre, mm, millimetre; PCL, posterior cruciate ligament; QT, quadriceps tendon; SD, standard deviation.

Logistic regression analysis identified A‐shape femoral notch (OR: 1.8; 95% CI: 1.1–2.9, *p* = 0.015), smaller LFC diameter (OR: 0.91; 95% CI: 0.85–0.98; *p* = 0.014) and female sex (OR: 1.9; 95% CI: 1.1–3.2; *p* = 0.012) as independently predictive of proximal versus non‐proximal femoral tunnel placement.

## DISCUSSION

The most important finding of this study was that BPTB autograft use was associated with more anterior femoral tunnel placement, while an A‐shape femoral notch, smaller LFC diameter and female sex were associated with more proximal femoral tunnel placement. The results of this study may be directly informative to surgeons about surgical and anatomical variables that influence femoral tunnel placement during primary ACLR.

The observed association between BPTB autograft use and more anterior femoral tunnel placement during primary ACLR may be explained by technical considerations unique to bone block fixation and passage during BPTB autograft ACLR. First, BPTB autografts are routinely secured with aperture fixation such as interference screws [31], which require adequate posterior bone stock to prevent posterior cortical blowout and compromise of interference screw fixation [22, 30]. In contrast, soft‐tissue autografts such as HT or QT autograft are often secured with suspensory devices [32], which can tolerate a more posterior femoral tunnel position as the risk for fixation compromise following posterior cortical blowout is reduced with the use of suspensory fixation [25]. Further, more posterior femoral tunnels during BPTB autograft ACLR may complicate the passage of the rigid bone blocks due to the acute transition angle between tibial and femoral tunnels [19, 20]. Given the above considerations, surgeons may intentionally position the femoral tunnel in a slightly more anterior position when using BPTB autografts and interference screw fixation, which explains the observed association in the present study. Second, when using BPTB autografts for primary ACLR, the tendinous surface of the autograft may be oriented towards the posterior aspect of the femoral tunnel to maintain an anatomic and posterior position of the tendon itself [3]. Therefore, it is possible that although the femoral tunnel itself is located in a more anterior position relative to other graft choices, the tendinous portion of the BPTB autograft is maintained with the anatomic femoral footprint of the ACL [9]. Nevertheless, the above factors unique to BPTB autograft ACLR are important intraoperative considerations while performing femoral tunnel drilling during primary ACLR.

An important finding of the present study was the association between an A‐shape femoral notch with more proximal femoral tunnel position during primary ACLR. While limited literature exists evaluating the association between femoral notch morphology and femoral tunnel position during ACLR, available evidence suggests more distal femoral tunnel placement may be achieved in patients with larger volume femoral notches [16], aligning with the results of the present study. Several prior studies have demonstrated that a stenotic or A‐shape femoral notch is an important risk factor for ACL injury and failure of ACLR [1, 2, 12, 29], likely explained by increased graft‐notch impingement as the knee is brought into full extension. The association between an A‐shape femoral notch and more proximal femoral tunnel placement may be explained by inadequate visualization of the lateral femoral notch wall during femoral tunnel drilling in patients with an A‐shape femoral notch, risking a discrepancy between achieved and intended anatomic femoral tunnel position [35]. Proximal femoral tunnel placement has been identified as an independent risk factor for ACLR failure in prior studies [6], therefore increased attention towards desired femoral tunnel placement may be warranted in patients with an A‐shape femoral notch [35].

Understanding surgical and anatomical variables that impact achieved femoral tunnel placement during primary ACLR has profound implications in preoperative and intraoperative decision‐making. Prior literature has demonstrated that, among a cohort of 221 international high‐volume ACL surgeons, there was significant variability in achieved versus intended femoral tunnel position [27]. The authors of the above study identified that the use of a malleable ACL ruler or fluoroscopy to localize femoral tunnel position resulted in improved accuracy of femoral tunnel placement compared to the use of an offset aimer or visual estimation of appropriate femoral tunnel position [27]. Therefore, in conjunction with the findings of the present study, it may be recommended to utilize more accurate tunnel localization strategies in patients undergoing BPTB autograft ACLR or with distal femur bony morphology that risks potential femoral tunnel malplacement. The cost of femoral tunnel malplacement during primary ACLR is high, and identifying surgical and anatomical variables that contribute to more anterior and proximal femoral tunnel placement may help reduce rates of ACLR failure, subsequent meniscal injury and long‐term progression of knee OA [6, 13, 14, 28].

There are several limitations to the present study. First, the inclusion of ACLR performed by six surgeons may introduce variability in the nuances of tunnel localization strategies that were not captured in this retrospective study [27]. However, all included surgeons are high‐volume ACLR and sports‐fellowship trained surgeons that utilize independent AM portal drilling for femoral tunnel creation, with the goal of achieving anatomic restoration of the native ACL footprint during primary ACLR. Second, as there is no accepted cutoff value delineating non‐anatomic femoral tunnel position in the P‐A and P‐D directions, femoral tunnel position groups in the present study were evaluated by comparing the most anterior and most proximal quartiles of femoral tunnels to the remainder of the cohort. However, it should be noted that, especially in the P‐D direction, the majority of femoral tunnels were placed distal to the commonly cited centre of the anatomic footprint (25% in the P‐A direction and 29% in the P‐D direction) [4]. Therefore, the present study's grouping of femoral tunnel position reflected the most anterior and proximal femoral tunnels within the study cohort, but may not reflect true non‐anatomic femoral tunnel position as it relates to the femoral ACL footprint. Nonetheless, the mean difference in femoral tunnel position between the study's groups in the P‐A and P‐D directions was 12% and 13%, respectively, which represents a clinically meaningful difference in femoral tunnel position between groups. Finally, the retrospective nature of the study with 47% of potentially eligible patients excluded introduces risk for selection bias in the present study.

## CONCLUSION

BPTB autograft use was associated with more anterior femoral tunnel placement, while an A‐shape femoral notch, smaller LFC diameter and female sex were associated with more proximal femoral tunnel placement during primary ACLR. For clinical relevance, these data may be useful to surgeons to identify demographic, surgical and anatomical variables that may influence femoral tunnel position during primary ACLR.

## AUTHOR CONTRIBUTIONS

All listed authors have contributed substantially to this work per ICMJE criteria for authorship. Literature search, study design and primary manuscript preparation were performed by Sahil Dadoo and Koji Nukuto. Data collection, analysis and interpretation of results were performed by Sahil Dadoo, Koji Nukuto, Efstathios Konstantinou and Jumpei Inoue. Final manuscript drafting and editing were performed by Jacob Hartline, Jonathan D. Hughes, James J. Irrgang and Volker Musahl.

## CONFLICT OF INTEREST STATEMENT

Jonathan D. Hughes: editorial board of *Knee Surgery, Sports Traumatology, and Arthroscopy* (*KSSTA*) and Annals of Joint; consultant for Smith & Nephew. James J. Irrgang: president, Board of Directors of Movement Science Media/Journal of Orthopaedic and Sports Physical Therapy. Volker Musahl: editorial board of *Knee Surgery, Sports Traumatology, and Arthroscopy* (*KSSTA*); Chair of Research for American Orthopaedic Society for Sports Medicine (AOSSM); consultant for Smith & Nephew. The remaining authors declare no conflict of interest.

## ETHICS STATEMENT

Ethical approval for this retrospective study was obtained from the associated university's Institutional Review Board (No.: STUDY19030196).

## Data Availability

The data that support the findings of this study are available on request from the corresponding author. The data are not publicly available due to privacy or ethical restrictions.
